# UV-B induction of the E3 ligase ARIADNE12 depends on CONSTITUTIVELY PHOTOMORPHOGENIC 1

**DOI:** 10.1016/j.plaphy.2015.03.006

**Published:** 2015-08

**Authors:** Lisi Xie, Christina Lang-Mladek, Julia Richter, Neha Nigam, Marie-Theres Hauser

**Affiliations:** Department of Applied Genetics and Cell Biology, University of Natural Resources and Life Sciences, Vienna, Austria

**Keywords:** Arabidopsis, ARIADNE12 (ARI12), CONSTITUTIVELY PHOTOMORPHOGENIC 1 (COP1), E3 ligase, Transcription, UV-B

## Abstract

The UV-B inducible *ARIADNE12 (ARI12)* gene of *Arabidopsis thaliana* is a member of the RING-between-RING (RBR) family of E3 ubiquitin ligases for which a novel ubiquitination mechanism was identified in mammalian homologs. This RING-HECT hybrid mechanism needs a conserved cysteine which is replaced by serine in ARI12 and might affect the E3 ubiquitin ligase activity. We have shown that under photomorphogenic UV-B, *ARI12* is a downstream target of the classical ultraviolet B (UV-B) UV RESISTANCE LOCUS 8 (UVR8) pathway. However, under high fluence rate of UV-B *ARI12* was induced independently of UVR8 and the UV-A/blue light and red/far-red photoreceptors. A key component of several light signaling pathways is CONSTITUTIVELY PHOTOMORPHOGENIC 1 (COP1). Upon UV-B COP1 is trapped in the nucleus through interaction with UVR8 permitting the activation of genes that regulate the biosynthesis of UV-B protective metabolites and growth adaptations. To clarify the role of COP1 in the regulation of *ARI12* mRNA expression and ARI12 protein stability, localization and interaction with COP1 was assessed with and without UV-B. We found that COP1 controls *ARI12* in white light, low and high fluence rate of UV-B. Furthermore we show that ARI12 is indeed an E3 ubiquitin ligase which is mono-ubiquitinated, a prerequisite for the RING-HECT hybrid mechanism. Finally, genetic analyses with transgenes expressing a genomic *pmARI12:ARI12-GFP* construct confirm the epistatic interaction between *COP1* and *ARI12* in growth responses to high fluence rate UV-B.

## Highlights

• ARIADNE12 (ARI12) is a functional monoubiquitinated E3 ubiquitin ligase.
• *CONSTITUTIVELY PHOTOMORPHOGENIC 1 (COP1)* regulates the UV-B induction of *ARI12*.
• A genomic *ARI12-GFP* fusion boosts growth in light and reduces tolerance to UV-B.
• *ARI12* is a target of a *COP1* controlled high fluence rate UV-B signaling pathway.

## 1 Introduction

The stratospheric ozone layer as well as oxygen, carbon dioxide and water vapor of the atmosphere absorb roughly 90% of the DNA damaging ultraviolet B (UV-B; 280–315 nm) radiation while most of the less harmful UV-A (315–400 nm) reaches the Earth's surface. Plants sense low fluence rate UV-B with the photoreceptor UV RESISTANCE LOCUS 8 (UVR8) which rapidly monomerize upon UV-B exposure and interact with the light signaling integrator CONSTITUTIVELY PHOTOMORPHOGENIC 1 (COP1) in the nucleus (Favory et al., 2009; Rizzini et al., 2011; Heijde et al., 2013; Huang et al., 2014). COP1 belongs to the large family of Really Interesting New Gene (RING) E3 ubiquitin ligases and acts in the dark as suppressor by targeting among others the photomorphogenesis promoting transcription factors ELONGATED HYPOCOTYL5 (HY5) and HY5 HOMOLOG (HYH) for degradation (Hardtke et al., 2000; Osterlund et al., 2000; Holm et al., 2002). In visible light COP1 is inactivated by the UV-A/blue light sensing CRYPTOCHROMES 1 and 2 (CRY1 and 2) (Lian et al., 2011; Liu et al., 2011; Zuo et al., 2011) and the far-red/red sensing PHYTOCHROMES A and B (PHYA and B) (Saijo et al., 2008) leading to the stabilization of the in the dark continuously degraded transcription factors. Upon supplemented UV-B light, COP1 is trapped in the nucleus by UVR8 allowing HY5 and HYH to activate genes that are responsible for the biosynthesis of UV-B protective metabolites and induce growth alterations (Ulm et al., 2004; Brown et al., 2005; Brown and Jenkins, 2008; Cominelli et al., 2008; Brown et al., 2009; Favory et al., 2009; Jenkins, 2009; Wargent et al., 2009; Stracke et al., 2010; Morales et al., 2013).

The *Arabidopsis thaliana* ARIADNE12 (ARI12) protein is a member of the RING-between-RING (RBR) family of E3 ubiquitin ligases (Mladek et al., 2003; Eisenhaber et al., 2007; Marín, 2010). ARI proteins were first identified in Drosophila (Aguilera et al., 2000). Arabidopsis has 16 members of this class including two pseudogenes. Although RBR proteins were thought to function as canonical RING E3s, recent studies on the human homolog of Drosophila Ariadne-1, HHARI, have shown that they employ a novel RING–HECT (Homologous to E6-AP Carboxy Terminus) hybrid mechanism which transfers ubiquitin to their targets (Wenzel and Klevit, 2012; Spratt et al., 2013). Classical RING-type E3 ligases mediate the transfer of ubiquitin from an E2 ubiquitin conjugating enzyme to the E3 associated substrate without being ubiquitinated themselves (Budhidarmo et al., 2012). For HECT-type E3 ligases, the ubiquitin is transferred first from the E2 conjugating enzyme to the E3 ligase and from there to the target lysine residue of the substrate (Metzger et al., 2014). Sequence comparison together with functional analyses identified a highly conserved cysteine at the C-terminus of the HECT- or RING2 domain that forms a thiolester bond between ubiquitin and the HECT- or RBR-type E3 ligases (Rotin and Kumar, 2009). Interestingly, this conserved cysteine is replaced by serine in the ARI12 protein. An experimentally introduced cysteine to serine substitution in HHARI has been shown to form a more stable ester with ubiquitin but the ubiquitin chain formation was not as efficient (Wenzel et al., 2011).

Under white light conditions *ARI12* is expressed at the detection limit and highly induced by UV-B (Mladek et al., 2003; Lang-Mladek et al., 2012). We have shown that under photomorphogenic, low fluence rate (LFR) of UV-B conditions, *ARI12* is a downstream target of the UVR8/HY5/HYH pathway. However under high fluence rate (HFR) of UV-B *ARI12* was partially regulated by HY5/HYH but UVR8 independent. Other photoreceptors such as PHYA and PHYB or the UV-A/blue light receptors PHOTOTROPIN 1 and 2 did not influence *ARI12s* expression. Nevertheless, CRY1 and 2 had a suppressive function on UV-B induced *ARI12* expression (Xie and Hauser, 2012). To date, nothing is known if and how the light integrator COP1 is regulating ARI12 expression and/or protein abundance.

Here, we investigated ARI12s E3 ligase activity and the possible monoubiquitination, a prerequisite for the RING-HECT hybrid mechanism. To clarify the role of COP1 in the regulation of ARI12, the expression was quantified at both, mRNA and protein levels, with and without UV-B as well as their protein interaction and ARI12s protein stability. Genetic analyses with genomic *pmARI12:ARI12-GFP* transgenes corroborate the role of COP1 in controlling ARI12 in white light, low and high fluence rate of UV-B. However COP1 is not the only regulator since low levels of ARI12 were still detectable in *cop1-4* mutants. Furthermore, our data suggest that the enhanced biosynthesis of ARI12 after UV-B exposure depends on a COP1 controlled high fluence rate UV-B sensing signaling pathway.

## 2 Methods

### 2.1 Cloning and transformation

For the recombinant production of GST-tagged ARI12 protein, cDNA was amplified with primer 1g05880_XhoI_F/R and cloned into pBADTOPO (Invitrogen, USA). After sequence confirmation the insert was transferred to the pGEX4T-1 vector using the XhoI restriction sites and transformed into the *Escherichia coli* strain DH5alpha. The GST-ARI8 and the HIS-AtUBC11 clones in strain BL21 (DE3) pLysS were a kind gift of Edward Kraft and Judy Callis (Kraft et al., 2005).

For generating the *pmARI12:ARI12-GFP* construct a 3754 bp genomic DNA fragment including the 1081 bp upstream region was PCR amplified with primers 1g05880pm_Xho_F and 1g05880_Xho_R and cloned into the pCR4TOPO sequencing vector (Invitrogen, USA). After sequence confirmation the total fragment was reamplified with primer SacI-F and SacI-R and inserted into the SacI site of the *pPZPMCSPUC211-GFP* binary vector. The *pPZPMCSPUC211-GFP* was generated by inserting the multiple cloning site of the *pUC-SPYNE* vector (Walter et al., 2004) into the SmaI and XbaI sites of *pPZP211* (Hajdukiewicz et al., 1994). GFP was amplified with primer GFP_BamHI and GFP_XbaI inserted into the respective sites in *pPZPMCSPUC211* to build *pPZPMCSPUC211-GFP*. The entire *gARI12-pPZPMCSPUC211-GFP* was sequenced and transformed through Agrobacterium mediated transformation into Col-0 using to the floral dip method (Clough and Bent, 1998). Transgenic seedlings were selected on MS medium supplemented with 100 mg L^−1^ kanamycin. Thirty one independent transgenic lines were tested for single insertion and nine were further analyzed for UV-B inducible GFP expression (Fig. 3A). The *pmARI12:ARI12-GFP* construct was introduced into *cop1-4* by crossing and homozygous *cop1-4* F3 lines were identified by their deetiolated phenotype under skotomorphogenic conditions. The presence of a homozygous transgene was determined on kanamycin medium. Sequence information of primers is provided in Supplementary Table 1.

### 2.2 Production of recombinant *UBC11*, *ARI12* and *ARI8*

The production of GST-ARI12 and GST-ARI8 was done in 100 mL YT-medium via 0.1 mM isopropylthio-β-galactoside (IPTG) induction at OD600 0.4 to 0.6 for 2 h at 37 °C. *E. coli* cells were harvested by centrifugation and lysed in 4 mL 1× PBS (137 mM NaCl, 2.7 mM KCl, 10 mM Na_2_HPO_4_, 1.8 mM KH_2_PO_4_) and sonication (7 × 30 s). After adding 80 μL Triton X-100, 15 min shaking and centrifugation for 20 min at 4 °C with 800 g the supernatant was added to 400 μL Glutathione-Sepharose 4B (Sigma–Aldrich) and incubated under rotation for 30 min at room temperature. The loaded GST-Sepharose was centrifuged for 5 min at 800 g and the pellet was washed three times with ice cold 1× PBS. The GST-tagged proteins were twice eluted with 2 × 800 μL of 10 mM reduced glutathione in 50 mM Tris–HCl pH 8 and centrifugation of 800 g at 4 °C. To each of the 200 μL aliquots, 80 μL sterile glycerol (87%) was added and stored at −80 °C until needed. Successful purification of the GST-tagged proteins was determined by 12.5% SDS polyacrylamide gels for electrophoresis (PAGE) and coomassie staining.

The 6xHis-tagged AtUBC11 was produced in 100 mL YT-medium supplemented with 100 mg L^−1^ ampicillin and 30 mg L^−1^ chloramphenicol and at OD600 0.4 to 0.6 induced with 0.1 mM IPTG for 3 h at 29 °C. Cell were harvested by centrifugation with 4000 g for 10 min, resuspended in 2 mL lysis buffer (10 mM imidazole in 1× PBS), loaded on a Ni-NTA Spin column (Qiagen, Germany), washed with 2 mL of 20 mM imidazole in 1× PBS and eluted with 300 μL of 250 mM imidazole in 1× PBS. Proteins were quantified with the Qubit method (Invitrogen, USA). Western blots (see below) with mouse anti-GST antibodies (Sigma–Aldrich) were used to confirm the presence and integrity of the recombinant fusion proteins.

### 2.3 Ubiquitination assay

Ubiquitination assays were carried out as described previously (Hardtke et al., 2002; Stone et al., 2005). Reactions (25 μL) containing 50 mM Tris–HCl, pH 7.5; 10 mM MgCl_2_; 0.05 mM ZnCl_2_; 1 mM ATP; 0.2 mM dithiothreitol; (DTT) 10 mM phosphocreatine; 0.1 unit of creatine kinase (Sigma–Aldrich); 50 ng of human recombinant E1 (Sigma–Aldrich); 6 μg of purified E2 AtUBC11; 2–8 μg of eluted GST-ARI12 or GST-ARI8 (positive control); and 2 μg ubiquitin (Sigma–Aldrich) were incubated at 30 °C for 3 h. Reactions were stopped by adding 5 μL of 6× loading buffer (0.3 M Tris–HCl pH 6.8, 0.6 M DTT, 12% [w/v] SDS, 60% [v/v] glycerol, 0.06–0.1% [w/v] Bromophenol blue), 3 min denaturation at 95 °C and loaded on a 10% SDS-PAGE followed by Western blotting using anti-ubiquitin antibodies (1:1000 in TBST; P4D1; Santa Cruz Biotechnology, Texas, USA). To confirm the presence of the recombinant GST-tagged ARI proteins and their monoubiquitination the blots were stripped and rehybridized with anti-GST antibodies (1:15,000) in 5% milk.

### 2.4 Plant materials and growth conditions

The *A. thaliana* accession Columbia (Col-0) was obtained from the *Arabidopsis* Biological Resource *Center* (ABRC, Ohio) and the mild *cop1-4* allele in Col-0 background was provided by Roman Ulm (McNellis et al., 1994; Oravecz et al., 2006). Seeds were surface sterilized with 5% sodium hypochlorite and sown on 1× Murashige and Skoog (MS) 1% agar media containing 4.5% sucrose (Hauser et al., 1995). After imbibition for 2 days at 4 °C in the dark, the nutrient agar plates were placed upright in a growth cabinet (RUMED Light Thermostats of Series 1000) for 11 days at 22 °C under continuous white light of 75 μmol m^−2^ s^−1^ photosynthetically active radiation (PAR).

Except for the narrowband low fluence rate (LFR) UV-B treatment conducted in Roman Ulm's lab (Favory et al., 2009), all UV-B experiments were carried out as described earlier (Lang-Mladek et al., 2012). UV-B treatment was either done with 12 day old seedlings on medium or with 28–30 day old soil grown plants. For RNA or protein analyses, entire seedlings or leaves were harvested at different time points, immediately frozen in liquid nitrogen and stored at −80 °C. For the chronic UV-B experiments, 11 day old seedlings were adapted to soil for 3–4 days under standard long-day white light (Philips F17T8/TL741) conditions of 140–150 μmol m^−2^ s^−1^ PAR and 22 °C prior of being exposed to a daily supplemental UV-B radiation of 4 μmol m^−2^ s^−1^, 1 h/day with unfiltered Philips TL20W/12RS broad band UV-B tubes. UV-B radiation started 3 h after the onset of the day/night cycle (16 h light/8 h dark). After 15 days growth was quantified by determining the rosette diameter and dry mass.

### 2.5 RNA analysis

Total RNA was isolated with TRIzol Reagent (MRC, Cincinnati, USA) according to the instructions. cDNA synthesis and quantitative real-time PCRs were performed as previously described (Karsai et al., 2002; Lang-Mladek et al., 2012). Primer pairs used for amplification are listed in Supplementary Table 1. Gene expression was calculated with the software of the RotorGene (Version 6.0) and Excel.

### 2.6 Plant protein analyses and Western blots

For protein extraction, plant tissue was pulverized in liquid nitrogen and for 100 mg plant powder 50 μL extraction buffer (25 mM potassium phosphate buffer pH 7.5, 1 mM ethylene-diaminetetraacetic acid (EDTA) pH 8.0, 1 mM DTT, 5 mM phenylmethylsulfonylfluorid (PMSF), Roche cOmplete Mini protease Inhibitor Cocktail (1 tablet for 10 mL buffer)) was added and vortexed for 1 min. The homogenate was centrifuged at 13,200 rpm for 10 min at 4 °C. The supernatant was transferred to fresh tubes, centrifuged again. After these two centrifugation steps the protein concentration of the supernatant was quantified with the Qubit^®^ 2.0 Fluorometer (Invitrogen). Since ARI12 is lowly expressed 75 μg of total proteins was needed to detect ARI12-GFP on Western blots. Proteins were denatured in 1× SDS loading buffer for 5 min at 95 °C, separated on 10% SDS PAGE and transferred onto a methanol pretreated polyvinylidene fluoride (PVDF) membrane (Roth). Tank electroblotting was done in a methanol free buffer (25 mM Tris–HCl pH 8.3, 192 mM glycine) at 28 V overnight (14 h) and 5 °C. To verify the uniform transfer, membranes were stained with 0.5% Ponceau S in 1% acetic acid. After blocking for 1 h with 5% skimmed milk in TBST (10 mM Tris–HCl pH 7.5, 150 mM NaCl, 0.1% (v/v) tween-20) membranes were briefly washed in TBST, incubated for 1 h with mouse monoclonal anti-GFP (1:2000, Roche) and mouse anti-actin (1:1000, Thermo Scientific) antibodies and after 3 times 10 min washes with TBST the secondary anti-mouse antibody conjugated to horseradish peroxidase (1:10,000, Pierce) was incubated for 1 h. Roti^®^-Lumin-plus substrate 1 and 2 (Roth) was equally sprayed on the membranes prior to exposure to X-ray films (Colenta, Mediphot X-90/RP Medical X-ray film). Protein abundance was quantified with the Gel Doc™ XR + imaging system (Bio-Rad) and normalized to actin.

### 2.7 Proteasomal degradation assay of ARI12-GFP

Seedlings grown on a solid MS medium for 12 days were transferred into liquid MS medium containing 0.5% DMSO with or without 100 μM MG-132 (Sigma–Aldrich) for 11 h and then exposed to white light supplemented with 4 μmol m^−2^ s^−1^ UV-B for 90 min. Samples were harvested before and 6 h after UV-B, rinsed three times with water and frozen in liquid nitrogen. Proteins were extracted and separated for Western blot analyses and detection of ARI12-GFP as described above. After stripping, the blots were rehybridized with anti-ubiquitin antibody to detect the accumulation of polyubiquitinated proteins caused by the inhibitory function of MG-132 on proteasomal degradation.

### 2.8 Yeast two hybrid protein–protein interaction analyses

Full length of ARI12 cDNA was cloned into the pCR4TOPO vector (Invitrogen, USA) and confirmed by sequencing. Inserts were transferred to the yeast-two-hybrid vectors pGADT7 and pGBKT7 (Clontech, USA) using the restriction sites introduced with the amplification primers (Supplementary Table 1) to create pGADT7-ARI12 and pGBKT7-ARI12. pGBK-UVR8 and pGAD-COP1 were kindly provided by Gareth I. Jenkins (University of Glasgow). pGBK-COP1 was generated from pGAD-COP1 by cloning COP1 into pGBK using *EcoRI* and *SalI/Xho1*. The AD and BD vectors were co-transformed into the *yeast* strain PJ69-4a (Clontech, USA) by the PEG/LiCl heat-shock method (Ito et al., 1983). The empty vectors pGADT7 and pGBKT7 were co-transformed as controls. Transformed yeast colonies were grown for 2–4 days at 29 °C on synthetic drop out (SD) medium (26.7 g L^−1^, Clontech) lacking leucine and tryptophane (−L/−T). To test UV-dependent interactions, transformants were transferred to fresh SD −L/−T plates and incubated for 20 h lid down in a growth chamber on a 32 °C heating block with 3 μmol m^−2^ s^−1^ UV-B (spectral energy irradiance was 0.13 W/m^2^ according to Flint and Caldwell's method (Flint and Caldwell, 2003)).

The strength of the protein–protein interactions was quantified with β-Galactosidase (β-Gal) activity assays using the ONPG (o-Nitrophenyl-β-d-galactopyranoside) method described by Guarente (Guarente, 1983). From each transformation three colonies were scraped off and resuspended vigorously for two minutes in 1 mL ice-cooled z-buffer (60 mM sodium phosphate, pH 7.0; 10 mM KCl; 1 mM MgSO_4_, and freshly added 50 mM 2-mercaptoethanol). The optical density (OD) was adjusted between 0.5 and 1 by adding z-buffer or cells. For lysis one drop of 0.1% SDS and two drops of chloroform were added to 800 μL yeast suspension and vortexed vigorously for 15 s. The β-gal reaction started after incubation at 30 °C for 15 min by adding 160 μL of ONPG-solution (4 mg mL^−1^). The reaction was stopped after 2 h with 400 μL of 1 M Na_2_CO_3_. Before measuring OD_420_ the samples were centrifuged at full speed for ten minutes. The β-Gal activity was calculated according to Miller's formula (Miller, 1972).

### 2.9 Subcellular localization of ARI12-GFP

To determine the subcellular localization of the ARI12-GFP fusion protein leaves of 25 days old plants were exposed to broad band HFR UV-B (4 μmol m^−2^ s^−1^) for 90 min and after 6 h mounted in tap water and observed under a 40× objective with a confocal laser scanning microscope (CLSM; Leica TCS SP2). GFP was excited using a laser at 488 nm and GFP emission was collected between 495 and 510 nm. To compare the GFP signal the photomultiplier and pinhole settings remained identical between the different samples. The GFP intensity was quantified with the help of the Leica LAS software and statistically evaluated in Excel. Wild type leaves were used as negative controls.

## 3 Results and discussion

### 3.1 *ARI12* is a functional E3 ubiquitin ligase with auto-monoubiquitinylation activity

**Fig. 1:**
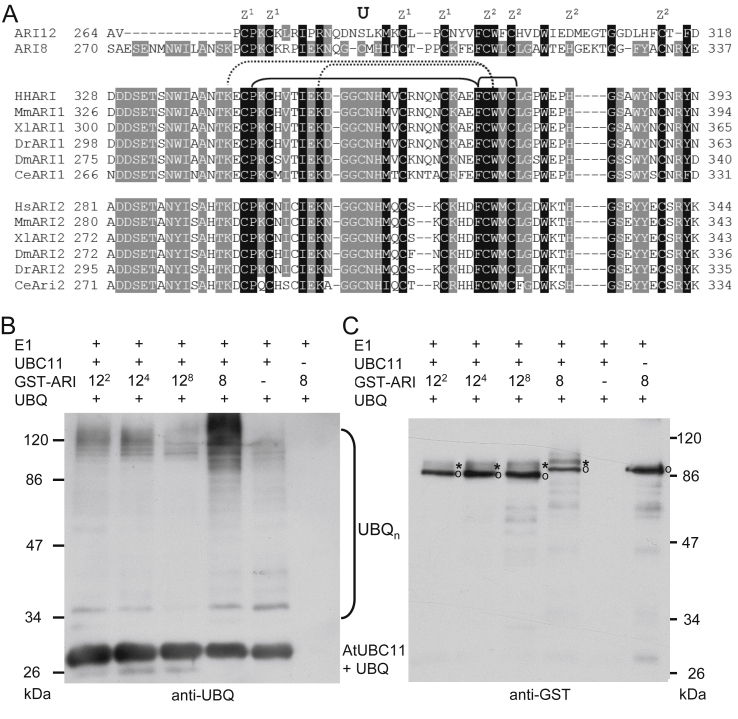
Sequence alignment of the RING2 domain and ubiquitination assays of ARI12 and ARI8. (A) Conserved residues between ARI12 and the structurally characterized HHARI and homologs from mouse (Mm), frog (Xl), zebra fish (Dr), fly (Dm) and worm (Ce). Cysteine and histidine residues coordinating the two Zn^2+^ atoms are marked with Z^1^ and Z^2^. The conserved cysteine forming the thiolester bond with glycine of ubiquitin is marked with U. Note that only in ARI12 this residue is serine. Conserved key contacts for F371 and W373 revealed in HHARI are marked with arches. Residues 100% and 90% identical between the sequences are highlighted with black and grey, respectively. (B, C) *In vitro* ubiquitination assays with GST-tagged full-length ARI12 and ARI8 mediate polyubiquitination. Omission of AtUBC11 and of GST-ARI8 resulted in a loss of protein polyubiquitination. Ubiquitinated proteins were visualized via Western blot analysis using ubiquitin (UBQ) (B) or anti-GST antibodies. Position and size of molecular markers are on the side of each blot. * mark the monoubiquitinated GST-tagged ARI proteins and open circle the GST-tagged ARI and UBC11 proteins. Superscript numbers indicated the increasing amount of GST-tagged ARI12 used in the assays.

Out of 14 putative E3 ubiquitin ligases of the ARIADNE family in Arabidopsis only for ARI8 the activity has been experimentally proven (Kraft et al., 2005; Stone et al., 2005). Although ARI12 belongs to the same subfamily as ARI8 it is the most distant member with variation in highly conserved residues in the RING1 and IBR (In Between Ring fingers) domain. Furthermore, ARI12 misses 13 residues in front of the RING2 domain (Mladek et al., 2003). Since the RING2 domain is the key portion for a RING-HECT hybrid mechanism (Spratt et al., 2013), we compared this domain of non-plant origin with that of ARI12 and ARI8 (Fig. 1A). The four highly conserved cysteines required for binding the first Zn^2+^ and the core FCW (phenylalanine, cysteine, tryptophane) motif for ubiquitin catalysis are present but one of the four cysteines/histidines for binding the second Zn^2+^ is missing (Fig. 1A). Furthermore, the single exposed cysteine used for the ubiquitin thiolester formation in HHARI is present in ARI8 at position 299 and substituted with serine in ARI12 at the corresponding position 281. However, serine as well as threonine can form equally well ester-bonds between the C-terminal glycine 76 of ubiquitin (Kravtsova-Ivantsiv and Ciechanover, 2012; McDowell and Philpott, 2013). Furthermore, the substitution of this cysteine with serine in HHARI was also able to form ester-bonds with ubiquitin but the ubiquitin chain formation was not as efficient (Wenzel et al., 2011). ARI12 is the only ARIADNE member of subfamily A and B which has a serine at this highly conserved position in RING2 (Mladek et al., 2003).

To determine if ARI12 is active and can be monoubiquitinated, the characteristic feature for a RING-HECT mechanism, we established an *in vitro* ubiquitination assay with recombinant and GST-tagged ARI12 or ARI8 protein, recombinant human E1 activating enzyme and Arabidopsis His-tagged E2 conjugating enzymes. The smear of high molecular weight demonstrates that ARI12 and ARI8 ubiquitinate proteins present in the assay (Fig. 1B and Supplementary Fig. 1). Furthermore the double band reveals the monoubiquitination of ARI12 and ARI8 (Fig. 1C), supporting that ARI12 and ARI8 are RING-HECT ligases and that this hybrid mechanism is conserved between plants and animals. However further experiments are needed to clearly prove that the serine at the position of the conserved cysteine in the RING2 domain of ARI12 is the target residue for the monoubiquitination. ARI12 provides a valuable system where the functional consequences of a serine to cysteine reversion can be explored *in planta* and consequently will help to advance the knowledge on the mechanism of this novel type of E3 ubiquitin ligases.

### 3.2 *ARI12* transcript levels depend on COP1 in white light and upon photomorphogenic and high fluence rate UV-B

**Fig. 2:**
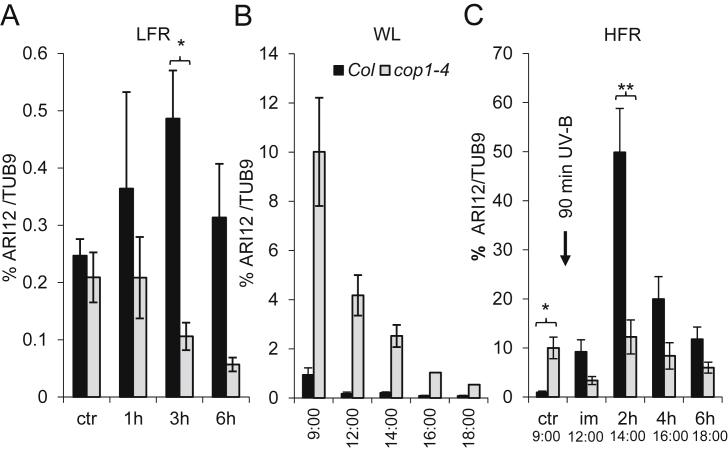
*ARI12* expression in *cop1-4* under white light, low and high fluence rate UV-B. (A) 12 day old seedlings treated with low fluence rate UV-B (LFR) for 1 h, 3 h and 6 h as described by Favory et al. (2009). (B) 25 day old plants under white light (WL) harvested at 9:00, 12:00, 14:00, 16:00, and 18:00. The harvesting time points are identical with that of the high fluence rate (HFR) UV-B experiments in (C). (C) 25 day old plants exposed to WL supplemented with HFR UV-B (4 μmol m^−2^ s^−1^) for 90 min. *ARI12* expression was normalized to the reference gene *TUB9*. ctr: before UV-B; im: immediately or 2 h, 4 h, 6 h after completing the 90 min UV-B exposure. Error bars indicate standard errors. * and ** correspond to p-values ≤0.05 and ≤0.001, respectively.

We have shown that *ARI12* is expressed at a very low level in leaves (Mladek et al., 2003) and strongly induced after exposure to UV-B radiation (Lang-Mladek et al., 2012). While the transcription of *ARI12* upon photomorphogenic UV-B relied on the UV-B photoreceptor UVR8 and the downstream transcription factors HY5 and HYH, under high fluence rates of UV-B the transcript levels were constitutively higher and increased further in an *uvr8* mutant background. The function of UVR8 depends on COP1 and we hypothesized that at least under low fluence rates and photomorphogenic UV-B conditions *ARI12* is regulated by COP1. Indeed, after photomorphogenic UV-B exposure *ARI12* expression was not induced rather decreased continuously suggesting that under low fluence rates *ARI12* depends on the classical UVR8/COP1/HY5/HYH signaling pathway (Fig. 2A).

While COP1 is a positive regulator in UV-B signalling it acts as repressor in visible light (reviewed in Lau and Deng, 2012). Thus if *ARI12* is downstream of COP1's repressive function *ARI12's* expression should be higher than in wild type in a *cop1-4* mutant background. Indeed, shortly after the night phase (1 h) the expression of *ARI12* in *cop1-4* was high and decreased continuously during the day (Fig. 2B). However, in white light with supplemented high fluence rate UV-B the expression of *ARI12* increased again in *cop1-4* but never reached the wild type level (Fig. 2C). These results imply that under white light only conditions COP1 is necessary to suppress the transcription of *ARI12* most probably via its downstream acting transcription factors HY5 and HYH. We previously showed that the *ARI12* promoter contains three G-boxes known to attract basic leucine zipper (bZIP) transcription factors such as HY5 and HYH. Furthermore, in low white light conditions *ARI12* expression was higher in the *hy5*/*hyh* double mutant compared to wild type (Lang-Mladek et al., 2012). The observation that *ARI12* is positively regulated by COP1 under both photomorphogenic and high fluence rate UV-B conditions fits with the protein abundance of ARI12 (next chapter).

### 3.3 *ARI12-GFP* localizes mainly in the cytoplasm 6 h after UV-B exposure

**Fig. 3:**
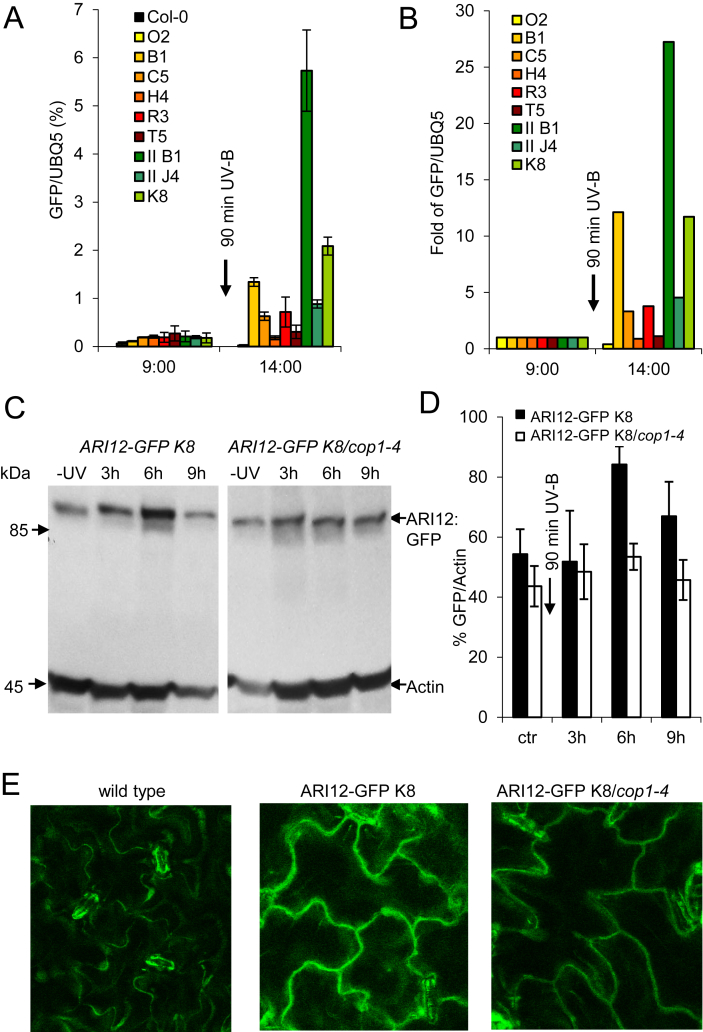
*GFP* expression upon HFR UV-B in *pmARI12:ARI12-GFP* lines are controlled by COP1. 25 day old plants were exposed to broad band HFR UV-B (4 μmol m^−2^ s^−1^) for 90 min (10:30–12:00). Leaves were harvested before (−UV) and at different time points after UV-B exposure. (A) *ARI12-GFP* expression was quantified with real-time PCR and normalized to the reference gene *UBQ5*. (B) Fold *ARI12-GFP* expression was compared with samples before UV-B exposure. (C) Western blots were probed with anti-GFP and anti-actin antibodies and (D) quantified and normalized to the actin loading control. (E) Detection of ARI12-GFP K8 in leaves of 25 day old plants around 6 h after a 90 min UV-B treatment. Pictures were taken on a CLSM.

Bioinformatic analyses suggested that ARI12 might be localized in the nucleus or shuttle between nucleus and cytoplasm (Mladek et al., 2003). Such a dual localization has been found for the human homolog HHARI (Ardley et al., 2001; Parelkar et al., 2012; Elmehdawi et al., 2013) and recently for ARI12 homologs in soybean (Chen et al., 2014). Since no specific antibodies could be generated for the determination of the subcellular localization of ARI12 and for the quantification of its COP1 dependent synthesis and stability upon UV-B, transgenic lines were established that contain a genomic *pmARI12:ARI12-GFP* construct regulated by the endogenous *ARI12* promoter. Out of 31 independent transformants 18 carried single insertions. For nine transgenic homozygous F3 lines the UV-B induced GFP expression was quantified with real-time PCR in a single experiment with three technical replicates (Fig. 3A,B). Most lines had a low background expression of the *ARI12-GFP* transcript. Thus the total *ARI12* transcript level (endogenous *ARI12* and *ARI12-GFP*) was increased in most of the lines (Supplementary Fig. 3). The two lines with the strongest UV-B inductions were *pmARI12:ARI12-GFP* IIB1 and *pmARI12:ARI12-GFP* K8 (Fig. 3A,B). As *ARI12* transcription is transient and peaks 2–3 h after UV-B exposure (Fig. 2A,C), we determined when the ARI12 protein reaches its highest level. As expected, the ARI12 protein peaked 3–4 h after the transcript maximum and 6 h after UV-B exposure (Fig. 3C). Similar to the transcriptional regulation the ARI12-GFP protein level was not significantly induced in *cop1-4* (Fig. 3D and Supplementary Fig. 2A,B). After determining the best conditions for ARI12-GFP visualization the K8 line was chosen for CLSM analysis. As shown in Fig. 3E 6 h after UV-B exposure ARI12-GFP accumulates mainly in the cytoplasm of leaf epidermal cells. Occasionally, ARI12-GFP was detectable in the nucleus independent of the UV-B treatment (Supplementary Fig. 4). The GFP signal in the *pmARI12:ARI12-GFP* transgene was clearly above the autofluorescence of the UV-B treated wild type control and present but to a lesser extent in the *cop1-4* background (Fig. 3E and Supplementary Fig. 3A). Quantification of the GFP signal revealed that in wild type after UV-B exposure ARI12-GFP was roughly 4 times more intense than in *cop1-4* or without UV-B treatment (Supplementary Fig. 4B,C). These data suggest that a significant fraction of the UV-B induced *ARI12* transcription and ARI12 protein abundance is controlled by COP1.

### 3.4 Inhibiting proteasomal activity does not alter the UV-B induced increase of *ARI12-GFP*

**Fig. 4:**
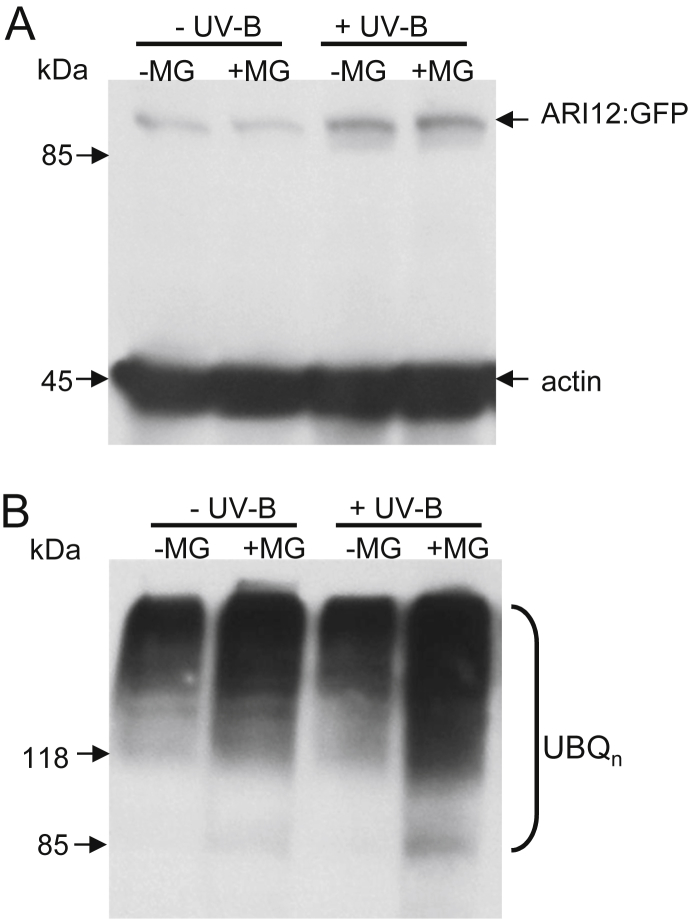
ARI12-GFP is not degraded by the proteasomal system. Seedlings grown on a solid MS medium for 12 days were transferred and incubated for 11 h in liquid MS medium supplemented with 100 μM MG-132 (+MG) in 0.5% DMSO or without (−MG). Samples before (−UV-B) and 6 h after (+UV-B) a 90 min UV-B exposure with 4 μmol m^−2^ s^−1^ UV-B were harvested and subjected to Western blot analyses. (A) ARI12-GFP and the loading control actin was detected using anti-GFP and anti-actin antibodies. (B) Western blots from A were stripped and probed with anti-ubquitin antibody.

While the transcription of *ARI12* upon high fluence rate UV-B is increasing more than 50-fold the induction at the protein level was only 1.5–2-fold and on the microscopic quantification 4-fold. The discrepancy between transcription and protein abundance cold potentially be explained by a constant degradation of ARI12-GFP through the proteasomal system. To test this hypothesis the proteasome was blocked with the inhibitor MG132 prior and during UV-B exposure. Western blot analyses showed that the abundance of the ARI12-GFP protein with and without UV-B was not changed in comparison to the no inhibitor controls (Fig. 4A). To prove that the MG132 treatment was functional the membrane was stripped and rehybridized with anti-ubiquitin antibodies. As shown in Fig. 4B, the smear of high molecular weight proteins was stronger in the inhibitor treated seedlings indicating that the MG132 was effective. Since the abundance of ARI12-GFP is not changed between inhibitor treated and untreated control it is unlikely that ARI12-GFP is degraded by the proteasomal pathway. These results imply that the abundance of the ARI12 protein might be regulated on a post-transcriptional/translational level or by MG132 insensitive proteases. In our previous *ARI* gene family characterization we identified PEST degradation signatures at the N-termini of several ARI proteins but not in ARI12 (Mladek et al., 2003). Evidence for degradation by other proteases is the instability of ARI12-GFP in extracts in the presence of protease inhibitors, in particular upon freeze thawing. Evidence for a post-transcriptional/translational regulation emerges from public microarray data, which indicate that under normal growth conditions *ARI12* transcripts are more abundant in non-polysomal fractions (www.ncbi.nlm.nih.gov/geo/tools/profileGraph.cgi?ID=GDS1382:261249_at) (Kawaguchi and Bailey-Serres, 2005). Furthermore, translational regulation related to light seems to be widespread (Bailey-Serres and Juntawong, 2012; Liu et al., 2013; Tsai et al., 2014). Future research will determine the molecular basis of the discrepancy between mRNA and protein abundance.

### 3.5 ARI12 does not interact with COP1 nor UVR8

**Fig. 5:**
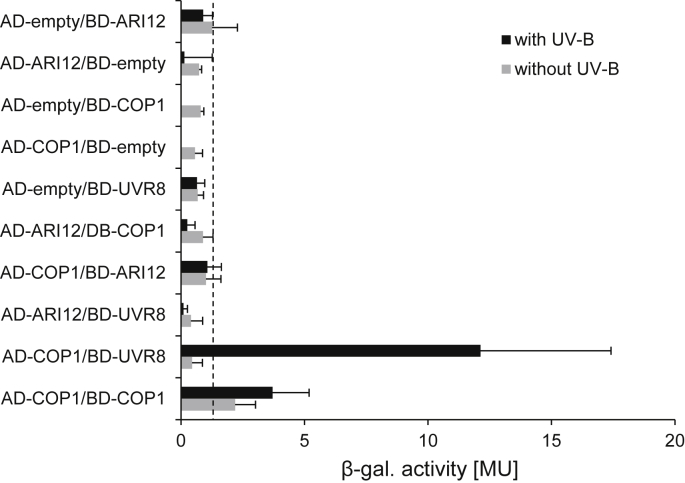
ARI12 does not directly interact with COP1 nor with UVR8. β-galactosidase (β-gal) activities of yeast two hybrid protein–protein interaction analyses between ARI12, COP1 and UVR8 without and after 16 h of 0.14 Wm^−2^ UV-B. Error bars correspond to standard errors.

It is known that RING domain containing proteins are frequently forming homo- and heterodimers (Metzger et al., 2014). The presence of other interaction domains such as the WD40 or coiled-coil domain in COP1 suggests the possibility of a direct interaction with ARI12 (Yi and Deng, 2005). One possibility to test for protein–protein interactions are yeast two hybrid (Y2H) assays. For COP1 this method has been proven to give reliable results for many interaction partners. Therefore, cDNA of *ARI12* was isolated and cloned into Y2H vectors. Interaction assays were conducted under white light and UV-B and quantified with β-galactosidase (β-gal) activity assays (Fig. 5). As positive control the published UV-B dependent interaction between COP1 and UVR8 was used (Favory et al., 2009; Cloix et al., 2012). Autoactivation of each construct was tested with empty vector cotransformations. While the UV-B induced interaction of COP1 and UVR8 could be confirmed, none of the interaction assays with ARI12 were above the background level. Only the COP1 dimer formation which has been shown to be mediated by the coiled-coil domain (Subramanian et al., 2004) was slightly enhanced under UV-B exposure (Fig. 5). Independent of UV-B ARI12 is able to homodimerize (Supplementary Fig. 5). Since COP1 is a member of the COP1–SUPPRESSOR OF PHYA-105 (SPA) complex we cannot exclude the presence or association of ARI12 in the approximately 700 kDa SPA complex, (Saijo et al., 2003). Other methods such as biochemical pull-downs and co-immunoprecipitation might exclude definitely such a direct interaction.

### 3.6 *ARI12* regulates UV-B dependent growth reduction

**Fig. 6:**
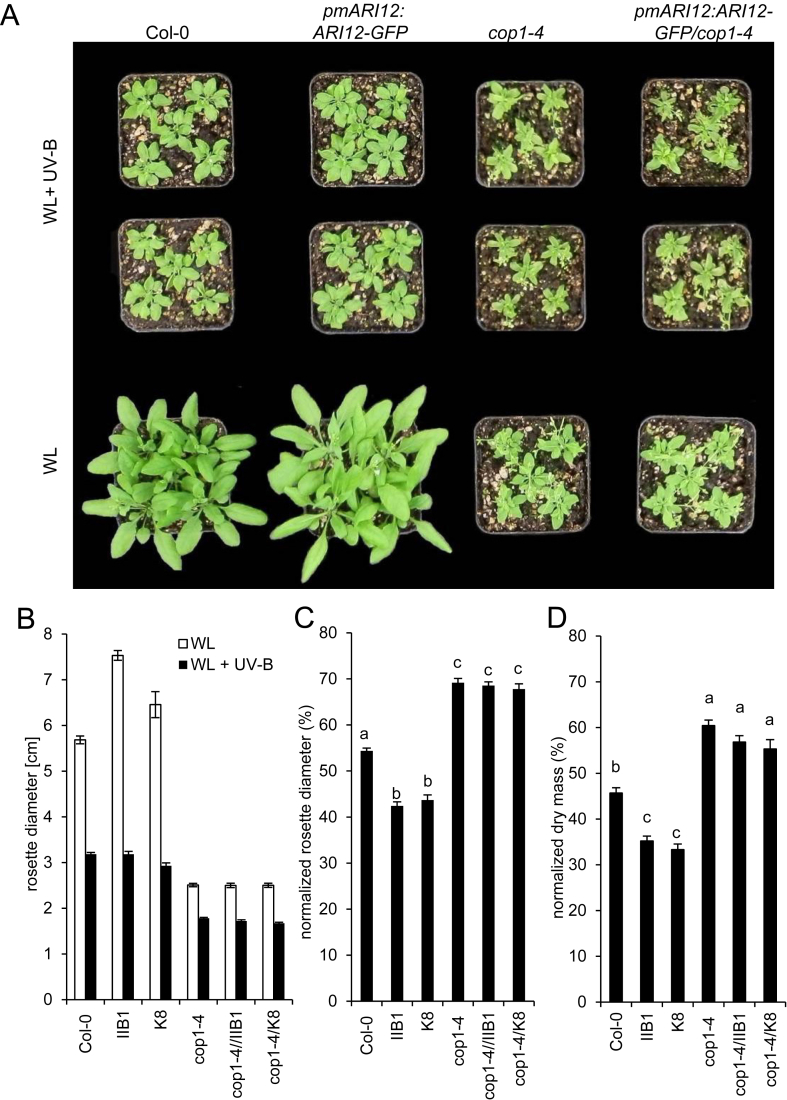
Phenotypes of the *pmARI12:ARI12-GFP IIB1* and *K8* lines and their *cop1-4* double mutants with and without chronic UV-B exposure. Approximately 14 day old plants were exposed to white light and daily supplemented for 1 h with HFR UV-B (4 μmol m^−2^ s^−1^) for 15 days. (A) Representative images of IIB1 plants after 15 days of UV-B exposure and the corresponding controls. The images of the K8 plants are in the Supplementary Fig. 6. (B) Rosette diameter of white light (WL) only and daily supplemented with UV-B (WL + UV-B). (C, D) Growth parameters normalized to the white light only controls of rosette diameters and dry mass. Error bars represent standard errors. The letters above the error bars represents significant differences between samples. Identical letters indicate no significant difference (p ≤ 0.05).

As expected, the total *ARI12* transcript abundance was higher in transgenic lines and probably due to the presence of additional genomic copies of *pmARI12:ARI12-GFP* (Fig. 3A and Supplementary Fig. 3). The variation between the transgenic lines was most likely due to the influence of the genomic insertion site of the added construct. As indicated above, we have chosen for further analyses two independent lines (IIB1 and K8) that showed the best UV-B inducibility. Under white light conditions the IIB line developed larger rosettes and leaves with elongated petiols while the rosette size of the K8 plants was more compact (Fig. 6A,B and Supplementary Fig. 6).

To determine the consequences of the *pmARI12:ARI12-GFP* expression during chronic UV-B treatments the vegetative growth was quantified after 15 days with a daily 1 h supplementation of 4 μmol m^−2^ s^−1^ UV-B. Since the rosette size varied between the wild type and the transgenes, all data were normalized with the non-UV-B white light control (Fig. 6). The rosette sizes of the two *pmARI12:ARI12-GFP* lines were reduced by 56.3–57.5% and for wild type by only 45.5% relative to control conditions (Fig. 6C). An even stronger trend was quantified for dry mass (Fig. 6D) supporting the idea that *ARI12* is involved in modulating growth upon UV-B exposure.

Since COP1 is regulating the UV-B induction of the endogenous *ARI12* gene as well as of the *pmARI12:ARI12-GFP* transgene, we asked if the missing UV-B induction of *ARI12* in *cop1-4* effects the long term growth acclimation to UV-B. Thus, the *pmARI12:ARI12-GFP* transgenic lines were crossed to *cop1-4* and the growth phenotypes were quantified in double mutants as described above. *Cop1-4* mutants developed small rosettes in white light and upon chronic UV-B exposure. In relation to the rosette size and dry weight of white light grown plants, the UV-B mediated reduction was, however, significantly less dramatic (Fig. 6C,D). As expected for an epistatic interaction between *COP1* and *ARI12*, addition of *pmARI12:ARI12-GFP* in the *cop1-4* mutant background abolished the reduced tolerance to UV-B. Thus, *COP1* is essential for the function of *ARI12* upon UV-B and the *COP1* and UV-B dependent induction of *ARI12* is involved in the UV-B induced growth retardation. In summary, the genetic experiments indicate that the abundance of *ARI12* needs to be strictly controlled and fine-tuned at the transcriptional and post-transcriptional level. Already small changes effect plant growth upon UV-B conditions.

Since ARI12 is a functional E3 ubiquitin ligase it is likely that ARI12 is involved in the degradation of a protein(s) that reduce growth upon UV-B. Thus, ARI12 candidate substrates might be proteins that exhibit a reduced abundance upon UV-B exposure. The proteome of maize leaves and ears changes rapidly in an organ and UV-B dosage specific manner (Casati et al., 2011). While in maize ears the majority (59 from 70) of proteins decreased in their abundance. This trend was not as clear in leaves (36 from 65). Protein stability regulations have been shown for key enzymes of the phenylpropanoid-biosynthesis, the phenylalanine-lyases (PALs) (Zhang et al., 2013) and the MAP KINASE PHOSPHATASE 1 (MKP1) (González Besteiro and Ulm, 2013). However these proteins are stabilized upon UV-B exposure and are constantly degraded under normal white light conditions. Therefore they are not likely substrate candidates for ARI12. Recently, Hayes et al. (2014) showed that UV-B stimulates the degradation of the PHYTOCHROME INTERACTION FACTORS (PIFs) PIF4 and PIF5. These transcription factors are regulating the activity of the growth hormones auxin and gibberellic acid. It has been suggested that the reduced abundance of PIF4 and PIF5 in UV-B decreases auxin activity, which limits elongation growth. These results were gathered under shade avoidance conditions and it needs to be seen if they play a similar role under high fluence rate UV-B conditions.

## 4 Conclusion

Here we analyzed the dependency of *ARI12* on the key light regulator COP1 and found that COP1 is essential for the transcriptional upregulation of *ARI12* after UV-B exposure. The observation that *COP1* activates *ARI12* transcription also at high fluence rates of UV-B independently of *UVR8* is a further indication of a yet unidentified UV-B sensing pathway. However transcription is not the only control of *ARI12*. Comparing the steady state level of *ARI12* transcript with that of the protein abundance revealed also a post-transcriptional fine-tuning of ARI12 which does not involve the proteasomal system and might be at the level of translation. Functional analyses showed that ARI12 is an active E3 ubiquitin ligase and very likely uses a RING-HECT hybrid mechanism to ubiquitinate its targets. Future analyses of ARI12 will address several compelling questions about its targets and their roles in the growth adjustment upon UV-B radiation.

## Contributions

L.X. did the expression analyses, crosses, genotyping, phenotyping and Western blots for ARI12 abundance. C.L-M. isolated RNA, produced cDNA, cloned and performed ubiquitinylation assays. N.N did the full length genomic fragment cloning of *ARI12* as well as the preparation and selection of the *pmARI12:ARI12-GFP* lines. J.R. performed the yeast two hybrid analyses and recloned *COP1* into the pGAD vector. M.-T.H. did the CLSM analyses, designed and wrote the manuscript together with L.X.
