# Supplementary material for: UV-B induction of the E3 ligase ARIADNE12 depends on CONSTITUTIVELY PHOTOMORPHOGENIC 1
Source: Plant Physiol Biochem. 2015 Aug;93:18–28. doi: 10.1016/j.plaphy.2015.03.006 (PMC4503874; doi:10.1016/j.plaphy.2015.03.006)
Supplement: Supplementary file 1 [file mmc1.doc]

**Supplemenatary Data**

UV-B induction of the E3 ligase ARIADNE12 depends on CONSTITUTIVELY PHOTOMORPHOGENIC 1

Lisi Xie, Christina Lang-Mladek, Julia Richter, Neha Nigam, Marie-Theres Hauser*

*β-Galactosidase activity assays for the ARI12-ARI12 interactions*

For each transformation, three fresh colonies were incubated in 5 mL liquid SD medium with 230 rpm for 2 days at 29 °C. One mL of this culture was added to 4 mL fresh liquid SD medium and incubated for 4 h. OD600 was determined at the start and 4 h later. Yeast cell were centrifuged with 14,000 rpm. The pellet was washed once with 1.5 mL buffer Z and resuspended in 300 μl buffer Z. Cell lysis was done by adding 25 μL chloroform and mixing for 15 sec. After pre-incubation for 10 min. at 30 °C, 100 µL of ONPG (4 mg.mL^-1^) were added and incubated with gentle shaking for 20 min. The reaction was stopped with 250 μL of 1 M Na_2_CO_3_, centrifuged at 10,000 rpm for 5 min. and the OD of the supernatant was measured at 420 nm. The β-galactosidase activity was calculated according to Miller’s formula (Miller, 1972).

**Supplementary Table I Oligonucleotide pairs used in the study**

| **Name** | **Sequence of primer pair**  **(5’- primer sequence- 3’)** | **use** |
| --- | --- | --- |
| 1g05880pm_Xho_F | ATCTCGAGGATCCACATGATCTAAC | GFP fusion with genomic ARI12 |
| 1g05880_XhoI_R | CTCGAGTTGATTACGGCTGAACCACT | GFP fusion with genomic and cDNA of ARI12 for Y2H |
| 1g05880_Xho_F | CTCGAGATGGATAATAATTCTGTAATCGGA | cloning cDNA of ARI12 for Y2H |
| SacI F | GAGCTCGATCCACATGATCTAAC | GFP fusion with genomic ARI12 |
| SacI R | GAGCTCTTTTGATTACGGCCTGACCCA | GFP fusion with genomic ARI12 |
| TH-ARI12-F-EcoR1 | TGAATTCatggataataattctgtaatcgg | cloning ARI12 into pGAD and pGBK for Y2H |
| TH-ARI12-R-EcoR1 | TGAATTCttattgattacggcctgaacc | cloning ARI12 into pGAD and pGBK for Y2H |
| GFP_BamHI | TGGATCCATGGTGAGCAAGG | for pPZP211MCSpUC |
| GFP_XbaI | ATCTAGATTTACTTGTACAGCTG | for pPZP211MCSpUC |
| ARI12pmBam_F | TAGGATCCACATGATCTAACGCTTTAG | promoter ARI12 for GUS fusion |
| ARI12pmBam_R | TTGGATCCGGTCTGACCATCTTC | promoter ARI12 for GUS fusion |
| GFP_Xho3 | CTGCTCGAGCTTGTACAGCTCGTC | for genotyping |
| GFP_Xho5 | AAGCTCGAGATGGTGACAAGGGCGA | for genotyping |
| GFP | CTGCTCGAGCTTGTACAGCTCGTC | for real-time PCR |
| GFP | AAGCTCGAGATGGTGAGCAAGGGCGA | for real-time PCR |
| ARI12 | ACGTATCGCAGCTTAAGTTCATCC | for real-time PCR |
| ARI12 | AATCCTTTGACGGTTCCTCCTCG | for real-time PCR |
| TUB9 | GTACCTTGAAGCTTGCTAATCCTA | for real-time PCR |
| TUB9 | GTTCTGGACGTTCATCATCTGTTC | for real-time PCR |
