# Supplementary material for: UV-B induction of the E3 ligase ARIADNE12 depends on CONSTITUTIVELY PHOTOMORPHOGENIC 1
Source: Plant Physiol Biochem. 2015 Aug;93:18–28. doi: 10.1016/j.plaphy.2015.03.006 (PMC4503874; doi:10.1016/j.plaphy.2015.03.006)
Supplement: Supplementary file 2 [file mmc2.pdf]

Supplemenatry Data

UV-B induction of the E3 ligase ARIADNE12 depends on CONSTITUTIVELY PHOTOMORPHOGENIC

Lisi Xie, Christina Lang-Mladek, Julia Richter, Neha Nigam, Marie-Theres Hauser

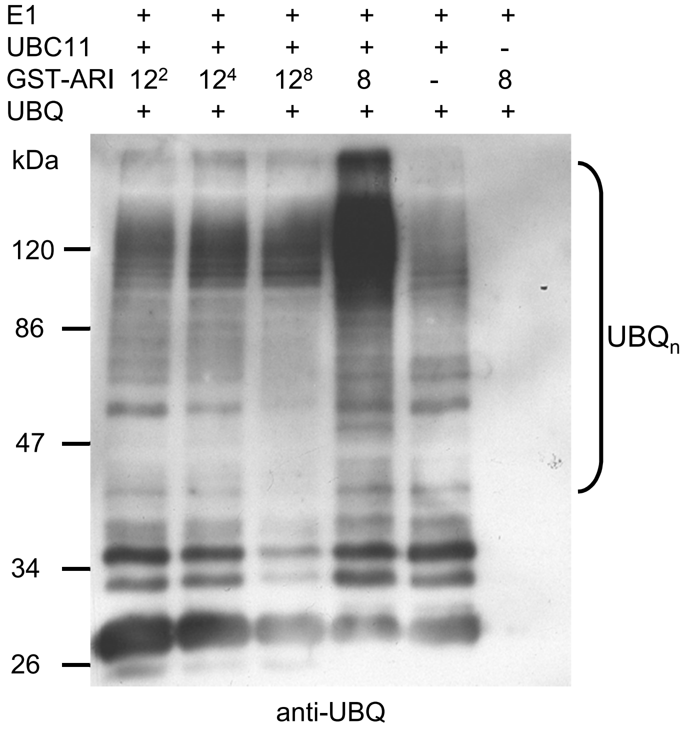

Supplementary Fig.1. Longer exposure of Western blot shown in Fig.1B

In vitro ubiquitination assays with GST-tagged full-length ARI12 and ARI8 mediate polyubiquitination. Omission of AtUBC11 reduce and of GST-ARI8 resulted in a loss of protein polyubiquitination. Ubiquitinated proteins were visualized via Western blot analysis using ubiquitin (UBQ). Position and size of molecular markers are on the side. Superscript numbers indicated the increasing amount of GST-tagged ARI12 used in the assays.

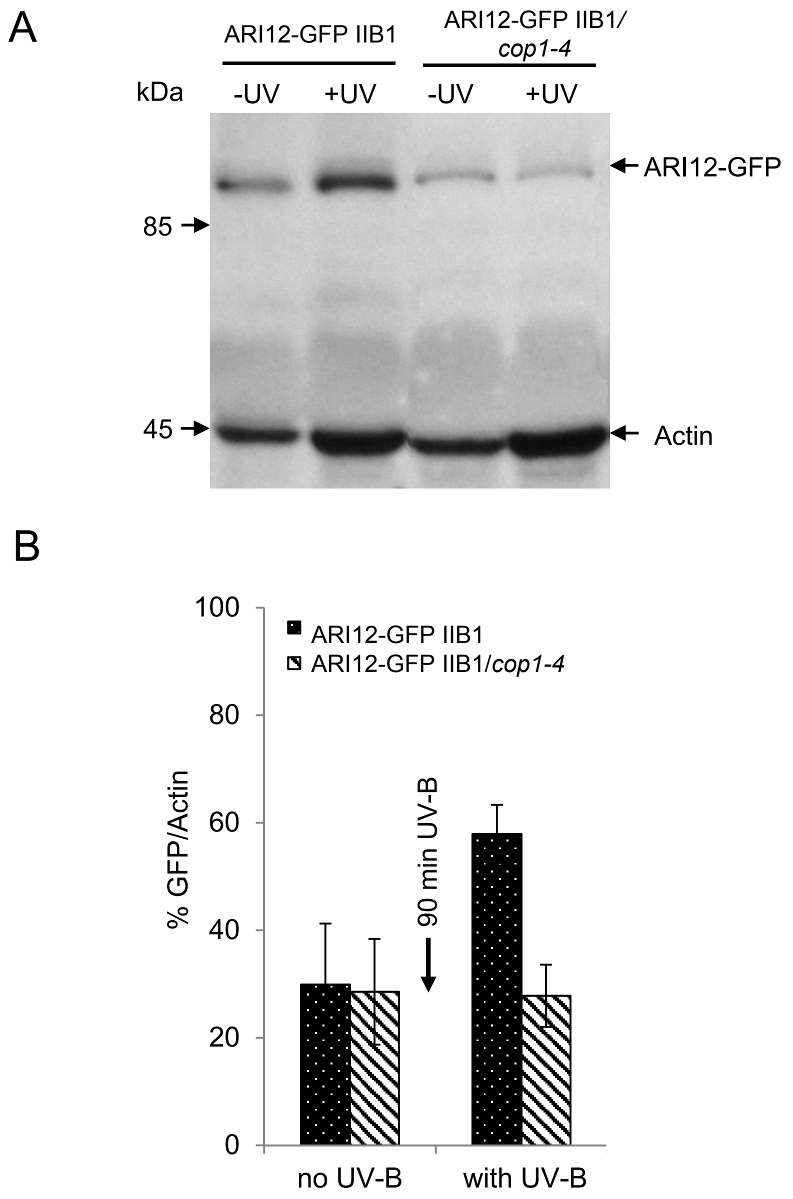

Supplementary Fig. 2. ARI12-GFP protein expression in *pmARI12:ARI12-GFP* IIB1/*cop1-4*

25 day old plants were exposed to broad band HFR UV-B (4 µmol.m^{-2}.s^{-1}) for 90 min. Leaves were harvested before (-UV) and 6 h after UV-B exposure.

(A) Western blots were probed with anti-GFP and anti-actin antibodies

(B) quantified and normalized to the actin loading control

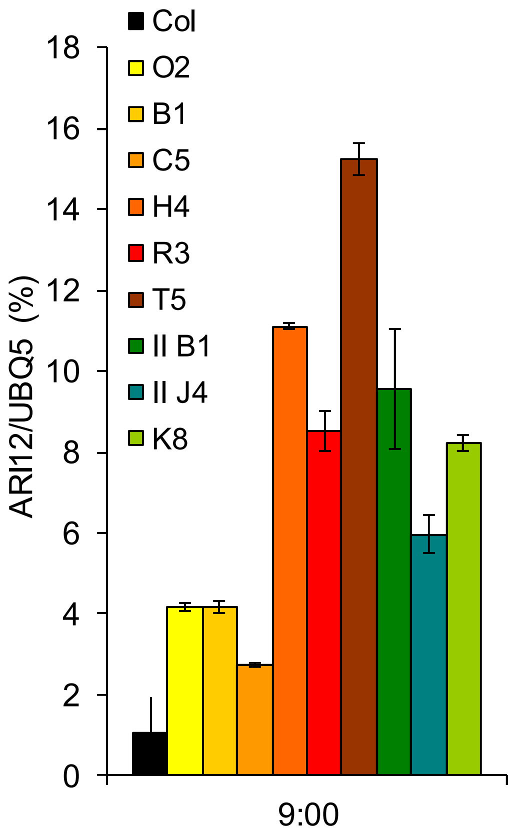

Supplementary Fig. 3. Total *ARI12* transcript abundance in transgenic *pmARI12:ARI12-GFP* lines

Total *ARI12* expression was quantified in leaves of 25 day old plants 1 hour after onset of white light with real-time PCR and normalized to the reference gene UBQ5. Error bars indicate standard deviations.

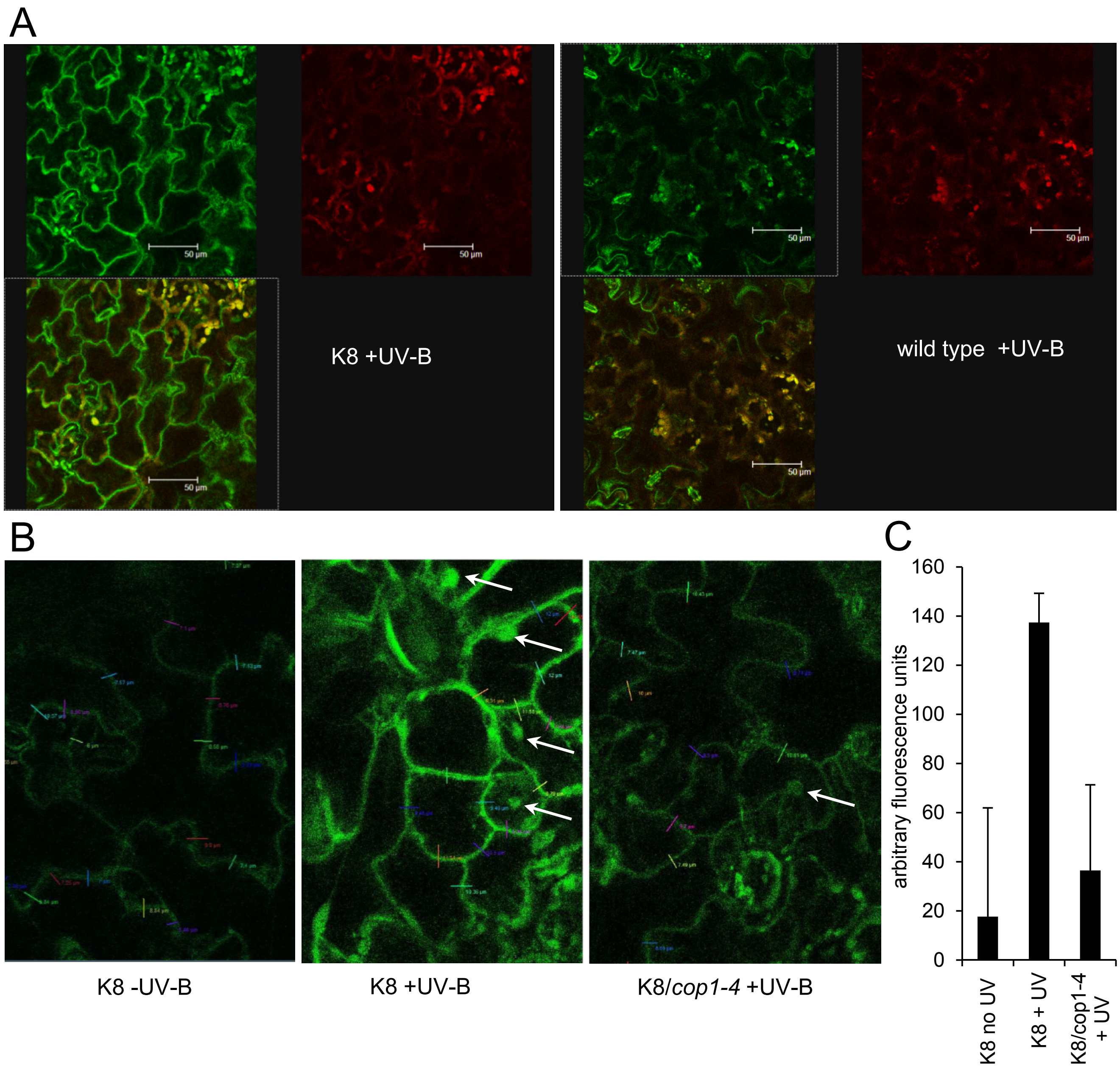

Supplementary Fig. 4. Cellular loaclization and quantification of the ARI12-GFP ***protein*** **expression**

25 day old plants were exposed to broad band HFR UV-B (4 µmol.m^{-2}.s^{-1}) for 90 min. Leaves were analysed in the CLSM before (-UV) and approximatly 6 h after UV-B exposure.

(A) ARI12–GFP expression of the K8 transgene 6 h after UV-B exposure and the autofluorescence. The green images represent GFP and the red chlorophyll detection, respectively. The lower green-yellow image is a merged image of the two green and red channels.

(B) Represenative pictures used to quantifiy the expression of GFP with and without UV-B treatment with and without the *cop1-4* mutant background.

(C) Summary of the image quantification supporting the UV-B inducibility and *COP1* dependence of the *pmARI12:ARI12-GFP* transgene K8.

White arrows indicate GFP-containing nuclei.

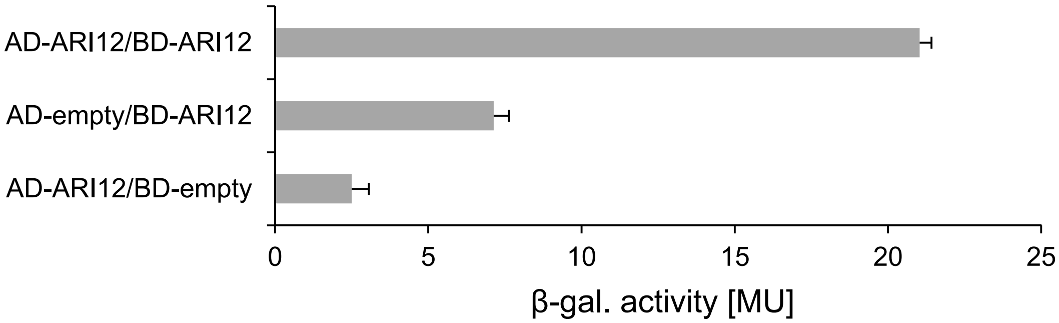

Supplementary Fig. 5. ARI12 is able to homodimerize

ß-galactosidase (ß-gal) activities of yeast two hybrid protein-protein interaction analyses. Error bars correspond to standard deviations.

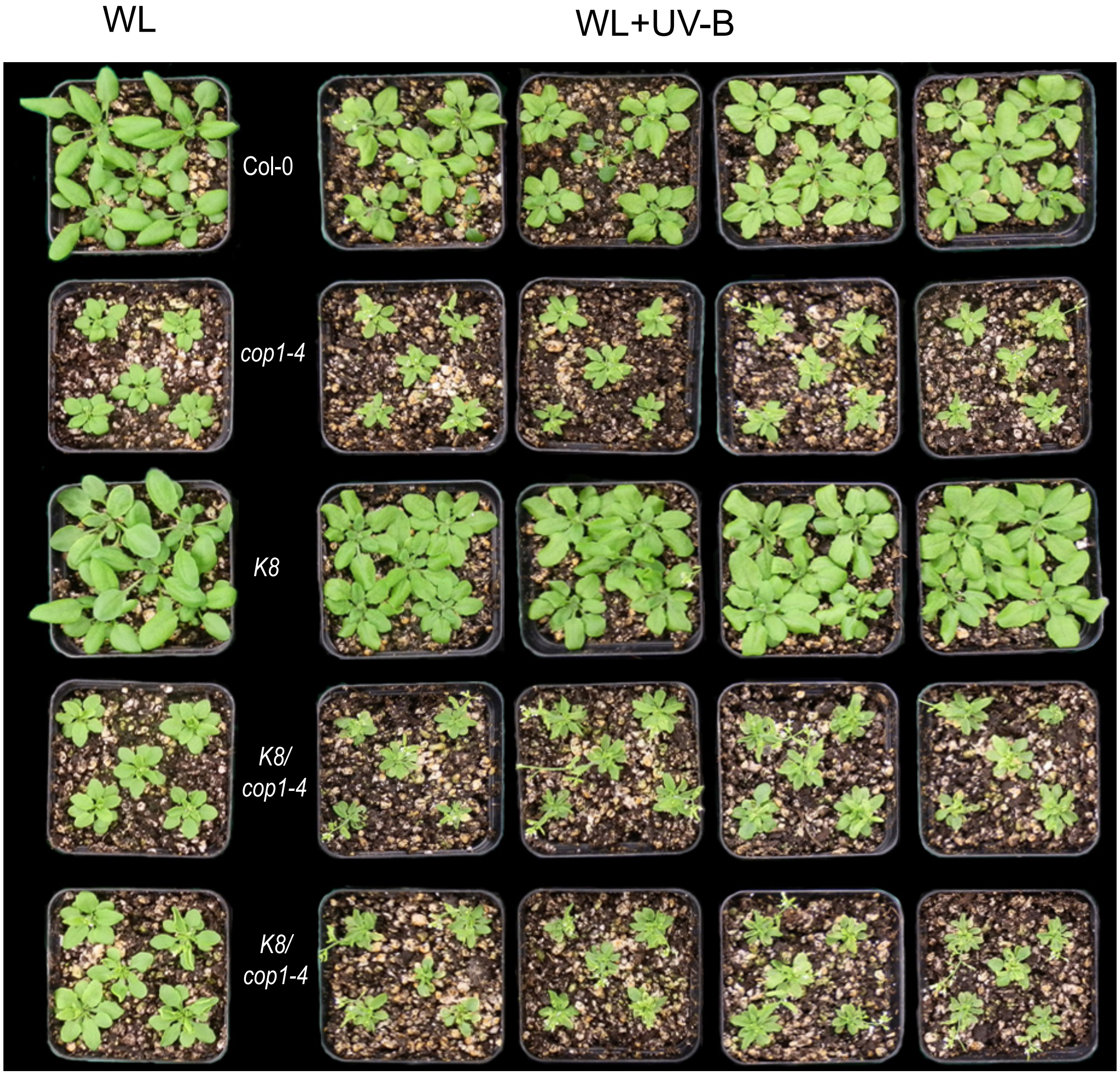

Supplementary Fig. 6. Rosette phenotype of the *pmARI12:ARI12-GFP* K8 line and their *cop1-4* double mutants with and without chronic UV-B exposure

Approximately 14 day old plants were exposed to white light and daily supplemented for 1h with HFR UV-B (4 $\mu mol.m^{-2}.s^{-1}$) for 15 days.

Representative images of plants after 15 days of UV-B exposure and the corresponding controls of the K8 transgene.
